# Improved Mechanical Amplification of Monolithic PZT and PZT Composite via Optimized Honeycomb Macrostructures

**DOI:** 10.3390/ma15227893

**Published:** 2022-11-08

**Authors:** Franziska Eichhorn, Julia Bytomski, Markus Gerauer, Ken-ichi Kakimoto, Tobias Fey

**Affiliations:** 1 Department of Material Science and Engineering, Institute of Glass and Ceramics, Friedrich-Alexander-Universität of Erlangen-Nürnberg, Martensstr. 5, D-91058 Erlangen, Germany; 2 Frontier Research Institute for Materials Science, Nagoya Institute of Technology, Gokiso-cho, Showa-ku, Nagoya 466-8555, Japan

**Keywords:** piezoelectric materials, ceramics-functional, ceramics-structural, ceramic composites, piezoceramic composites, cellular solid, functionalization, strain amplification

## Abstract

Honeycomb-based, modular composites with a relative density of 0.3948 and a slenderness ratio *L_ges_/t* of 6.48 were fabricated on PZT building blocks connected with a PZT-filled phenyl silicone resin. The macro- and micro-structure, phase composition, and the interface between the two materials were analyzed by SEM and image analysis techniques. The mechanical *in-plane* strain response was determined with uniaxial compression tests and the *transversal* piezoelectric strain response was determined by applying an electric field. These deformations were analyzed by a 2D digital image correlation analysis to calculate the mechanical strain amplification of monolithic and composite PZT lattice structures. Compared to bulk PZT, the piezoelectric strain amplification in the Y-direction |*a_y_^piezo^*| was higher by a factor of 69 for the composite and by a factor of 12 for the monolithic cellular PZT lattice, when it was assumed that the ratio of the deformation of the bulk material to bulk material was 1. The mechanical amplification of the composite lattices increased up to 73 and that of the cellular PZT lattices decreased to 12. Special focus was given to the fracture behavior and the interface of the PZT/PZT-filled phenyl silicone resin interface.

## 1. Introduction

Cellular solids can be found in nature [1] and are adapted in molecular or macroscopic dimensions for technical applications. Due to their excellent strength- and stiffness-to-weight ratio, energy absorption capacity, and enhanced stiffness against shear deformation they are used in aerospace, naval, sporting, and automotive applications [2,3,4,5,6,7,8]. According to Nielson et al. [9] and Safari et al. [10], these structures or lattices are “3-1-connected composites” and exhibit anisotropic behavior of thermal, mechanical, or electrical properties. The functionalization of cellular solids, e.g., the piezoelectric effect, has a high potential for further applications and an enhancement of material properties [2,11]. Chiral and hexagonal honeycombs were made from a shape memory alloy ribbon material to improve the potential for thermally-activated adaptive and deployable structures [12]. Sanada et al. [13] combined a piezoelectric actuator with an auxetic honeycomb-type design mechanical amplifier and improved the maximum actuation strain per unit height by 3% (=410 µm for a height of 14 mm).

Active piezoelectric, cellular solids with an internal architecture are suitable for many hardware applications. Passive piezoelectric lattice types with their unique and tailor-made microstructural and topological properties have shown an increased resistance to shear deformation in out-of-plane mode. Therefore, Iyer et al. [5,14] used finite element (FE) models and investigated the relations of piezoelectric honeycomb structures as a function of the relative density and connectivity. They determined the influence of the deformation mode (longitudinal/out-of-plane, transversal/in-plane) and the lattice geometry (regular and anisotropic honeycombs, and tetragonal and triangular reference structures) on the deformation behavior. While the transverse behavior depends mainly on the cellular structure and connectivity, the longitudinal behavior is almost linearly influenced by the relative density [14]. The piezoelectric constants can be optimized by tailoring the cellular design. Based on this research, Eichhorn et al. [15,16] analyzed the mechanical and pseudo-piezoelectric deformation behavior of modular, honeycomb-based cellular solids by FE simulations. The lattice structures consisted of modular units (building blocks), which were connected by a piezoelectric passive epoxy and were excited individually. Compared to monolithic bulk material, a maximum strain amplification of 12.1 was obtained. This depends on Young’s modulus, the excitation mode, and the slenderness ratio.

In addition to FE simulations [14,15,16], experimental investigations on active lattice structures made of pure piezoelectric ceramics, polymers, or composite materials are reported [2,6,17,18,19]. A basic piezoelectric cellular, auxetic lattice structure based on PZT laminates (single thickness 0.53 mm) was reported by Fey et al. [2]. The mechanical and piezoelectric strain responses of auxetic honeycombs were determined by uniaxial compression load as well as an electric field perpendicular to the lattice plane [2]. The strain amplification in the Y-direction showed an enhancement factor of 34.0 for mechanical deformation and 30.3 for piezoelectric deformation [2].

In this work, honeycomb-based, modular and cellular structures, were described. Composite lattices were made by connecting PZT building blocks with a piezoelectric active PZT-filled phenyl silicone resin (Eichhorn et al. [20]). The use of reactive or active fillers to modify or influence the properties of preceramic polymers before or after their transformation has been known since the study of Greil [21]. Eichhorn et al. [20] have already reported on PZT as a piezoelectric active filler for preceramic polymers. This work combines preliminary work consisting of FE simulations of lever structures [15] with the practical development of a new type of resin that resulted in composite lattice structures.

The focus of this research was to analyze the mechanical amplification behavior and, most especially, the influence of the interface between building blocks and the PZT-filled resin on the fracture behavior. The results show that the processing caused a gradient structure [22,23,24,25,26,27] and a textured surface [22,23,28,29,30,31,32].

## 2. Materials and Methods

### 2.1. Sample Production

**Figure 1:**
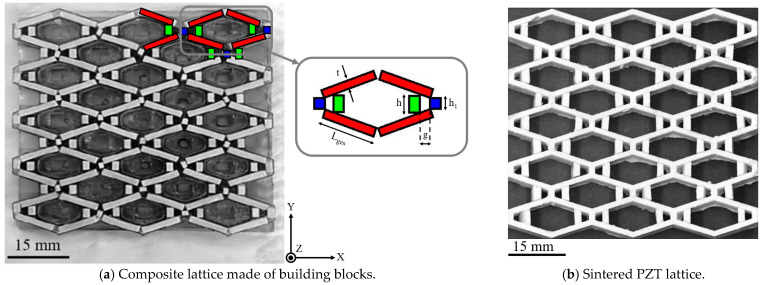
Fabricated PZT lattice structure constructed from ceramic building blocks in 3D-printed negative form with schematic drawing of modular unit cell (**a**) and reference structure made from the same PZT injection mold (**b**).

A soft PZT powder Pb(Zr_x_Ti_1–x_)O_3_ with *x* = 0.44 − 0.52 and spray granules with an average grain size of d_50_ = 1.8 µm (NCE51, Noliac Group, Kvistgaard, Denmark) was used to fabricate the cellular composite according to the design of Eichhorn et al. [16,33] with 23 unit cells. The ceramic building blocks were produced using a PZT injection mold with a filler content of 48.3 vol-% [16,34,35] and cast into three different 3D silicone forms [35,36] generated by casting 3D-printed positive forms (Printing material “DL260”, 3D printer “028 J”, both DWS Systems, Zane, Italy) (Figure 1). Filling and cooling were carried out at 120 °C and with a cooling rate of 1 K min^−1^ in a tempering furnace (“Type K1252”, Heraeus Holding GmbH, Hanau, Germany) to reduce thermal induced stress. Before deforming, the top and bottom of the building blocks were ground by silicone carbide paper with different grits (“Waterproof Silicone Carbide Paper FEPA”, P#320 to P#1200, Struers GmbH, Ottensoo, Germany). Debinding (600 °C, dwell time 2 h) and sintering (1250 °C, dwell time 2 h) were carried out in a ZrO_2_-MgO powder bed (“Zirconium(IV)oxide”, Thermo Fischer (Kandel) GmbH, Karlsruhe, Germany and Magnesium oxide, VWR International GmbH, Darmstadt, Germany). To reduce lead evaporation, sintering was carried out under a PbO-atmosphere (PbO-powder: Blei(II)-Oxid gelb (99%), Lot code 1064203 12704153, Fluka Chemika; furnace: Type “KL10/13”, Thermoconcept, Dr. Fischer GmbH&Co.KG, Bremen, Germany). The three building blocks types (Figure 1a) had the dimensions of height *h*_1_ 0.91 mm × sample thickness 4.87 mm × strut thickness *t* 1.38 mm (type 1, blue), height *h* 2.10 mm × sample thickness 4.86 mm × strut thickness *t* 1.20 mm (type 2, green), and length *L_ges_* 7.16 mm × sample thickness 4.82 mm × strut thickness *t* 1.27 mm (type 3, red), and were set into the 3D-printed negative forms (Printing material “DC700”, 3D printer “028 J”, both DWS Systems, Zane, Italy) (Figure 1b), and connected with the phenyl silicone resin polymer “Silres H62 C” (Polysiloxane, Wacker Chemie AG, Munich, Germany) with PZT filler (PZT-filled silicone resin). The gap *g* between the two small building blocks with heights *h* and *h*_1_ had a width of 1.50 mm. The production of this filled silicone resin is described below. In comparison to [16,33], the connecting phase of epoxy resin was replaced by PZT-filled silicone resin without conversion or further processing to the ceramic, and to improve the singular piezoelectric properties of the complete structure.

A reference structure with identical geometrical dimensions was fabricated from monolithic PZT ceramic (Figure 1b) consisting of the same PZT injection mold used for the building blocks of the composite lattice structures. For this purpose, the 3D-printed negative forms “DC700” were filled with the injection mold, demolded, and sintered in a powder bed analogously to the building blocks.

The PZT filler for the silicone resin was coated twice with C_12_H_28_O_4_Zr (Zirconium(IV) n-propoxide, 70% w/w in n-propanol, Alpha Aesar, Johnson Matthey GmbH, Karlsruhe, Germany) and afterward dried at 300 °C. At this temperature, C_12_H_28_O_4_Zr transformed into ZrO_2_, which should protect the PZT powder from reactions with the Pt-catalyst of the phenyl silicone resin.

The coated PZT powder was hydrophobized by stearic acid (0.1 vol-% Number 27403, Honeywell Riedel de Haen^®^, Seelze, Germany) and hexane (14.5 vol-% n-Hexan, Merck KGaA, Darmstadt, Germany) according to Eichhorn et al. [20]. A 70 vol-% silicone resin fraction and a 30 vol.-% PZT fraction as filler were mixed in a vacuum stirrer for 30 min and filled between the building blocks into the 3D-printed negative forms, fabrication mentioned above. To generate a pore-free connecting phase, the form was degassed in a vacuum chamber and afterward pre-cured at 150 °C. After deforming, the connected lattices were cured at 220 °C for 5–6 h.

To measure the piezoelectric properties, metallization was done by using self-adhesive Aluminum foil (“tesa Aluminum Tape”, Tesa, Norderstedt, Germany) which was attached by a thin layer of touch-dried conductive silver paste (Acheson, Silber DAG 1415 mit Pinsel, Plano GmbH, Wetzlar, Germany) to improve the connectivity. The reference materials were coated with a silver paste (“solderable paste for screen printing 61901113”, Ferro GmbH, Freigericht, Germany) and fired at 700 °C without any change in the crystallographic properties. Both types of polarization were carried out in a silicone oil bath (Wacker AK20, Wacker Silicones, Wacker Chemistry AG, Meinich, Germany) at 130 °C and an electric field of 2 kV mm^−1^. The optimal poling conditions and the computation of the effective poling field were estimated by the Kura Kawa theory of Furukawa et al. [37,38] in 1976, which has previously been applied to PZT-filled silicone resin [20]. The Kura Kawa theory [38] describes the effective electric field intensity that acts on the in-polymer distributed piezoelectric active particles.

### 2.2. Characterization

The microstructure of the porous samples was determined by SEM (FESEM, Helios 4 NanoLab 600i FIB Workstation, FEI, Hillsboro, OR, USA) in electron backscattered mode. The true density of ceramic building blocks and filled polymer were measured by helium pycnometer (”Micromeritics Accu Pyc II, Typ 1340, Gas Pycnometer“, Micromeritics GmbH, Mönchengladbach, Germany) and attained 7.47 g·cm^−3^ (94% fractional density). The theoretical relative density of the materials and the cellular solids was calculated by the ratio of geometrical and true density values. By creating a 3D model, a theoretical volume was obtained using RP software (VisCam RP, Marcam Engineering Software Informer Inc.) that could be used to determine the theoretical density. The different phases of the cellular composites were determined by the open-source image software “ImageJ” (“ImageJ”, Version 1.51 g).

The longitudinal piezoelectric constant *d_33_* and the capacitance *C_p_* (relative permittivity *ε_r_*) were measured on five samples each of the composite and each lattice type with a piezoelectric testing system (“Piezo-Meter PM 300”, Piezo Test, London, UK) at *f* = 110 Hz. The values obtained for dense PZT reference samples made of PZT injection mold (porosity of about 6%) were *d^bulk^*_33_ = 325 pC N^−1^, *d^bulk^*_31_ = −140 pC N^−1^, and relative permittivity *ε^bulk^_r_* = 1195 [2], respectively.

The piezoelectric strain response is the result of the lattice middle unit cell deformation in the X- and Y-direction (*ε*_1_ and *ε*_2_) determined on five samples of each lattice type. It was initiated by a constant (DC) electric field of *E*_3_ = +566 V mm^−1^ applied in the Z-(poling) direction of the lattice structure. The 2D images were obtained by optical recording (“Dynax 7D”, Konica Minolta Inc., Tokyo, Japan, and lens: “Life-size attachment No. 2019552”, 17–70 mm F2 8-4 DC Macro, OS HSM, Sigma Deutschland GmbH, Rödermark, Germany) analyzed with the software Veddac 6.0 (Chemnitzer Werkstoffmechanik GmbH, Chemnitz, Germany) to calculate *ε*_1_ and *ε*_2_ from the non-deformed and deformed states, as described in [10,17,39]. The mechanical and piezoelectric strain response *d_ij_* were calculated according to Equation (1).

(1) $$\varepsilon_{j}=d_{ij}E_{3}$$

Details of the evaluation are described in Fey et al. [2]. The factor of strain amplification corresponds to the ratio of deformation of the composite to the reference material (PZT bulk material) [15,16]. Because of pure lateral dynamic excitation in the X-direction, the focus was set to the higher deformation in the Y-direction. Young’s modulus of the reference material and composites was determined by the oscillatory impulse excitation method (Buzz o Sonic 4.0, BuzzMac International, LLC, Portland, ME, USA) on rectangular cross-sections, which were placed on wedge-shaped foam bearings, and via software.

Strength *σ_b_* and deformation *ε*_2_ of the five samples of each type were determined from compression tests (feed rate 1 mm/min, 500 N load cell, “Instron 5565”, Instron Deutschland GmbH, Pfungstadt, Germany). To guarantee a homogenous load distribution, the structures were placed upright in a PVC holder (“PVC-hart-Platten dunkelgrau”, König GmbH Kunststoffprodukte, Gilching, Germany). The in-plane deformation was recorded by a high-speed-camera perpendicular to the lattice structures fixed at Y = 0 (“SpeedCam MarcoVis EoSens” and “MotionBLITZ Cube7” and “SpeedCam Basis Software v1.11.28”, Mikrotron GmbH, Unterschleissheim, Germany) and analyzed with Veddac software.

## 3. Results and Discussion

### 3.1. Structure Analysis

The porosity of bulk PZT and filled silicone resin was determined from geometric and true density *ρ^PZT^*, *ρ^filled silicone resin^*), and the results are shown in Table 1. Based on this data and Equation (2), the theoretical density of the composite material $\rho_{theo}^{composite\;material}$ as PZT building blocks plus PZT-filled silicone resin was calculated from the phase fractions of PZT *Φ^PZT^* (74.3%) and polymer *Φ^filled silicone resin^* (25.6%).

(2) $$\rho_{theo}^{composite\;material}=\rho_{}^{PZT}\Phi_{}^{PZT}+\rho_{}^{filled\;silicone\;resin}\Phi_{}^{filled\;silicone\;resin}$$

This corresponds to a phase fraction of 28.5% for the PZT building blocks and 11.5% for the PZT-filled silicone resin, including cavities, which account for about 60%.

Eichhorn et al. [20] showed that PZT spray granules without a ZrO_2_ coating break during the hydrophobization and homogenization process. The ZrO_2_ coating acts on the one hand as mechanical protection against crushing and on the other hand as a reaction barrier with the Pt-catalyst in the silicone resin. Based on the SEM analysis, it was determined that more than 71% of the spray granules are mechanically intact.

**Figure 2:**
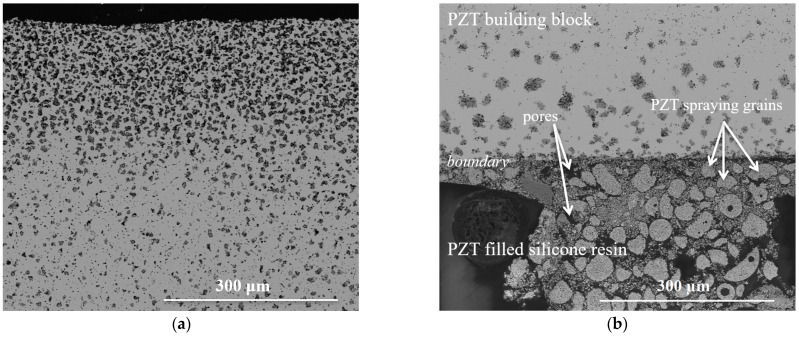
Mechanical connectivity of the interface between PZT building blocks and PZT-filled silicone resin. The line indicates the interface between the PZT blocks and filled resin. (**a**) Surface of a sintered PZT building block. (**b**) Interface between PZT building blocks and PZT-filled silicone resin.

Figure 2a shows the surface of a sintered PZT building block without connection to another. The porosity is decreased in the core but increased on the surface. The interface between the building block and PZT-filled silicone resin is shown in Figure 2b.

Of particular interest is the influence of the manufacturing process on the interface. The PZT building blocks have a gradual porosity on the surface (5.92%, Figure 2a), which is caused by PbO evaporation and is desirable in this case and belongs to stochastic surface textures [22,23,24,25,26,27]. According to McBain et al. [40], pores and gradient structures are the oldest and simplest way to create a mechanical interlock (Figure 2b). A structured surface or roughness are parameters that affect the strength of bonded joints, as it increases adhesion by mechanical interlocking and leads to an increase in the contact area between the joining part (PZT building block) and the adhesive (PZT-filled silicone resin) [41,42,43]. In addition, due to the 3D printing of the positives for the silicone molds, the PZT building blocks have a textured surface [22,23,28,29,30,31,32] with linear undulations analogous to [22,23,32]. The influence on the properties was investigated by Biggemann et al. [23] and confirmed by Naat et al. [32,44]

The novel combination of the 3D printing process and injection molding leads to a significant enhancement of mechanical interlocking in the interface, which reduces the mechanical deformation behavior during compression tests.

**Figure 3:**
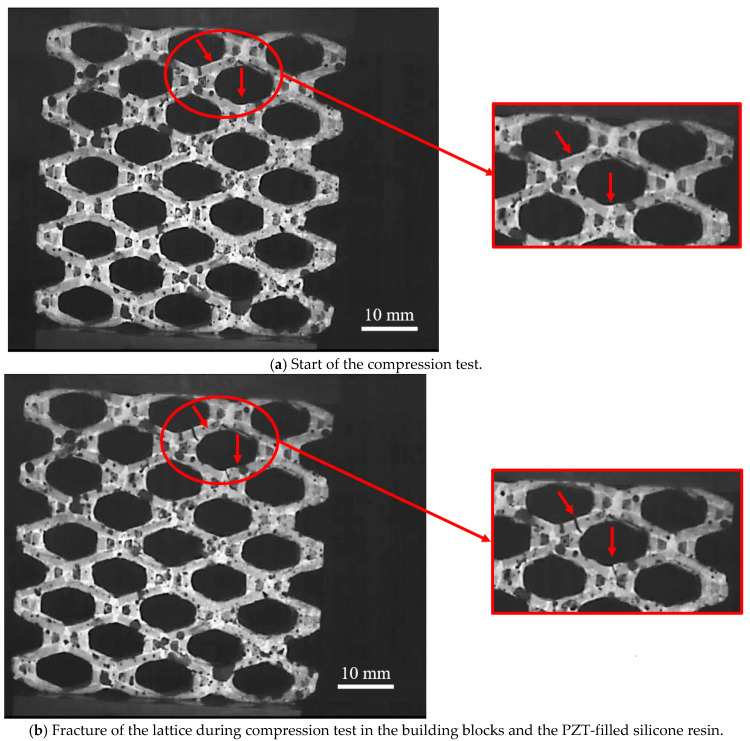
Different stages of the compression test: (**a**) compression test start and (**b**) at fracture of the lattice in the building blocks and the PZT-filled silicone resin. In the figure, one sample is shown as an example.

Mechanical properties, such as Young’s modulus and compressive strength, determined by compression tests and theoretical calculations are listed in Supplementary Material Table S1. The details of the calculations can also be found in the supplementary material. The properties of the bulk PZT showed higher values than the PZT-filled silicone resin or the composite lattice consisting of PZT building blocks and PZT-filled silicone resin due to the presence of pores or a polymer phase. Figure 3 shows the fracture behavior of one of the five samples of the composite lattices. The compression test was a destructive process, so the tests couldn’t be repeated. The intact lattices (Figure 3a) deformed and cracks appeared (Figure 3b). The fractures, and, thus, the failure of the composite lattice structure, occurred in the upper rows of the structure and the building block or the PZT-filled silicone resin (Figure 3b). The interface remained intact (arrows in Figure 3a). This deformation was additionally analyzed by FE simulation, not shown here. The struts of the upper unit cells deformed while the unit cells on the base (Y = 0) remain almost in their initial state. Due to this discovery, the deformation and strain amplification were determined at the middle unit cell to reduce the influence of the boundary area. The distance between the interface and the crack was measured in each case. For the cracks within the PZT-filled resin, a distance between 468 ± 52 µm and 658 ± 55 µm was found, and for the cracks within the PZT building blocks between, the distance was between 3562 ± 42 µm and 3926 ± 148 µm. Therefore, the damage to the interface could be excluded.

The interlocking increased the mechanical properties at the interface and improved the performance and mechanical deformation of the whole composite lattice structure compared to PZT lattices, as can also be seen in Table S1 in the supplementary material [22,23]. This agrees with tensile stresses analyzed by FE-simulated stress behavior and strain amplification of cellular 2D grating and actuator structures [15,16,33] and corresponds to the crack during the compression tests.

Due to both manufacturing processes, pores are present in the PZT injection mold and the PZT-filled silicone resin. Compared to the reference material bulk PZT injection mold (*ε_r_* = 1195, *d*_31_*^PZT^* = 325 pC N^−1^, *d*_31_*^PZT^* = *d*_32_*^PZT^* = −140 pC N^−1^), the PZT lattices and the composite lattices showed an improvement in mechanical amplification in the X- and Y-directions |*a_x,y_^piezo^*| caused by piezoelectric excitation, and the values can be taken from Table 2. For PZT lattices and composite structures, the maximal mechanical amplifications were increased by a factor of 18.40 and 69.40, both detected in the Y-direction and when it is assumed that the ratio of the deformation of the bulk material to bulk material is 1 (Equation (S1) in the supplementary materials).

In general, increased porosity led to a reduction in the piezoelectric properties in the form of the relative permittivity and piezoelectric coupling coefficients. A porosity of 6% reduced the piezoelectric properties by up to 37% when compared to the information provided by the manufacturer (Table 2). In all cases, and following, the relative percent change was calculated from the difference between the final value and the initial value divided by the initial value.

Compared to the reference material (bulk PZT injection mold), the out-of-plane properties of the PZT lattice structures in the form of the relative permittivity *ε_r_* and *d*_33_ were reduced by a maximum of 41%, but the in-plane deformation *d*_31_ and *d*_32_ increases by up to 826.3% and 1737.0%. This was similar for the composite structures made of PZT-filled silicone resin and PZT building blocks: *ε_r_* and *d*_33_ decreased by 93.8% and 88.6% and *d*_31_ and *d*_32_ improved by 1414.3% and 1739.0%.

The PZT-filled silicone resin had very low piezoelectric properties (*ε_r_* = 2.87, *d*_33_*^PZT^* = 0.31 pC N^−1^, *d*_31_*^PZT^* = *d*_32_*^PZT^* = −0.08 pC N^−1^) due to the small amount of non-connected PZT filler (30%) and an additional 3% porosity. Comparing these values to the values previously reported (*d*_31_ = −0.12 pC N^−1^, *d*_33_ = 0.27 pC N^−1^, [20]), the improvement in the reduction of the piezoelectric properties seen in this work is caused by a higher homogeneity and the retained ZrO_2_-coated spray grains. The thin zirconia layer isolates the PZT spray grains from the electric field and reduces the response of the PZT-filled silicone resin.

The reduction of the singular material piezoelectric properties by 89%, especially in the out-of-plane direction, increases the piezoelectric deformation/amplification in the in-plane by about 1800%. Likewise, the modular design and the interlocking interface and junctions made of PZT-filled silicone resin bring additional toughness and mobility.

### 3.2. Mechanical Amplification

**Figure 4:**
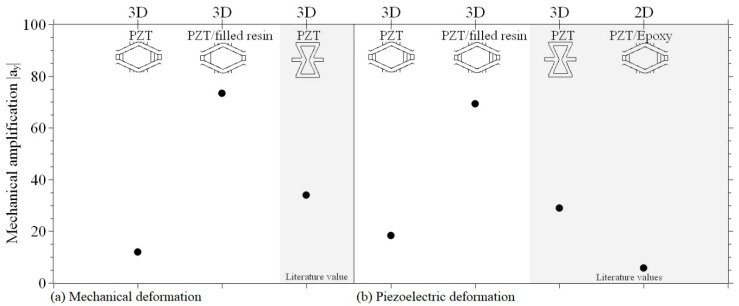
Mechanical amplification as a ratio of the deformation of cellular structures and bulk material as a function of mechanical (**a**) and piezoelectric (**b**) excitation.

The mechanical amplification in theY-direction |*a_y_^mech^*| caused by compression is shown in Figure 4. |*a_y_^mech^*| was calculated as the ratio of the lattice deformation to the mechanical bulk material deformation *ε^reference,mech^*. The details of the calculation can be found in the supplementary material. The calculation of the mechanical strain amplification is done according to Equations (S2)–(S4) in the supplementary materials.

The mechanical properties, such as Young’s modulus or compressive strength of the reference bulk PZT, were decreased by introducing a structured design (Table S1 in the supplementary materials). The Young’s modulus was reduced by 90% for the PZT lattice and by 47% for the composite lattices, and the compressive strength by 85% and 95%, respectively. Comparing the two types of lattices (PZT and PZT-PZT-filled silicone resin composite), the mechanical amplification |*a_y_^mech^*| was improved due to the elastic part of the silicone resin (Figure 4). This increased the deformation towards plastic behavior and led to |*a_y_^mech^*| increasing from 12 to 73 (Table S1 in the supplementary materials), an increase of 508%. Compared to the PZT lattice, the compressive strength of the composite lattice decreased by 65% due to the joints made consisting of PZT-filled silicone resin. Young’s modulus increased by 426%.

Based on literature data of lasered PZT foils with auxetic structure design [2], and the results of this work, it can be derived that the strain amplification is structure-based. Auxetic lattices are inverted honeycomb-based structures with re-entrant cell geometry. These had an angle of −25° and were made of laminated PZT foils fabricated by tape casting. The thickness was 0.53 mm. Fey et al. [2] reached a strain amplification of 30.3 in the X-direction and 29.0 in the Y-direction. Comparing the two different structures made of bulk PZT showed that the auxetic structure had a slightly increased mechanical amplification. However, the comparison cannot be differentiated because the material and the component height (0.53 and 5.00 mm) differ too much. Therefore, the mixture of material and structural influence cannot be resolved.

### 3.3. Piezoelectric Strain Response

The *in-plane* lattice strains were measured in the middle unit cell of the lattices according to Fey et al. [2], and the longitudinal piezoelectric constants and strain amplification were calculated according to Equations (S1) and (S5) in the supplementary materials. The results are listed in Table 2 and Figure 4. Comparing PZT cellular solids and composite lattices with the same structure design, the material influence on the piezoelectric strain amplification could be determined and revealed. |*a_x_^piezo^*| was improved by 211.8%, from 9.30 to 29.00, and |*a_y_^piezo^*| by 277.2%, from 18.40 to 69.40. This is due to the modular design of the composite structure with joints made of flexible PZT-filled silicone resin, just as with the amplification under mechanical loading.

As mentioned above, the structure reported in [2] also showed a mechanical amplification of auxetic lattices under an applied electric field. Comparing the two different structure types of PZT bulk material, an increase in |*a_x_^piezo^*| of 225.8% and |*a_y_^piezo^*| of 57.6% was determined for the auxetic structure. However, a material influence can be completely rejected due to the different manufacturing methods of casting and injecting.

The influence of different structure parameters such as slenderness ratio, and the high potential of 2D honeycomb-based structures, have already been studied in detail by experiment and FE simulation [15,16]. The maximum strain amplification in the Y-direction was 5.7. Therefore, dimensionality and an increase in mass in the Z-direction have a positive effect on the mechanical strain amplification. Due to the many differences between the practical implementation (3D) of the ideal FE models (2D), composite type, and fixation type, a comparison was waived.

## 4. Conclusions

In this work, 3D piezoceramic pure PZT lattices and modular composite lattices were fabricated from PZT building blocks connected by an interface of PZT-filled silicone resin [1]. The PZT-filled silicone resin was prepared with zirconia-coated PZT powder to reduce reactions with the Pt-catalyst of the resin, which may lead to bubbles. Due to the fabrication methods, a combination of 3D printing and injection molding, a new type of interlocking was generated. This reinforced the interface by a combination of stochastic (pores) and textured (linear waves) surface design. Compared to a bulk reference, the singular mechanical and piezoelectric properties, such as Young’s modulus or d_33_, were reduced by 94% due to the structured design of the lattices. Conversely, the mechanical strain amplification |*a_x,y_*| was improved independently of piezoelectric and mechanical excitation. A maximum |*a_y_*| was reached in the Y-direction, and was 73.0 for compression mode and 69.4 for applied electric field mode. The modular and composite design with joints made of PZT-filled silicone resin induced an improvement in the mechanical amplification of 508% compared to PZT references. Additionally, the influence of the structure design could be emphasized. Compared to auxetic structures made of laminated PZT tapes, which showed an increase in |*a_x,y_*| (30.3 or 29.0) [2] of up to 225.8%, PZT lever structures showed an amplification of 9.3 to 18.4. However, the influence of the material could not be excluded in this case. The influence of the structure and especially of the slenderness ratio was also identified in prior research [2,15].

## Figures and Tables

**Table 1:** Density and the average porosity of the PZT building blocks, the PZT-filled silicone resin, and the produced composite material.

Material Unit	True Density g cm^−3^	Geom. Density g cm^−3^	Porosity vol%
Bulk PZT	7.94	7.47	5.92
Filled silicone resin	3.27	3.17	3.00
Composite material as PZT Building Blocks plus PZT-filled silicone resin	6.74	6.36	

**Table 2:** Piezoelectric coupling coefficients of bulk PZT, PZT lattices, PZT-filled silicone resin, composite lattice structures, and literature values [2]. Using these values, the mechanical amplification was calculated. In addition, the relative percentage changes of the piezoelectric properties of the lattice structures in relation to the bulk material were calculated and listed. The mechanical amplification was calculated using the average of the determined deformation.

Material	Relative Permittivity		Piezoelectric Coupling Coefficients						Mechanical Amplification According Supplementary Equation (S5)			
	*ε_r_*		*d*_31_		*d*_32_		*d*_33_		|*a_x_^piezo^*|		|*a_y_^piezo^*|	
		*%*	pC N^−1^	*%*	pC N^−1^	*%*	pC N^−1^	*%*		*%*		*%*
Bulk PZT	1195 ± 30		−140.00 ± 3		−140.00 ± 3		325.00 ± 23		1		1	
PZT lattice	709 ± 62	−40.7	−1296.80 ± 40	+826.3	−2571.80 ± 39	+1737.0	320.00 ± 15	−1.5	9.30	+830.0	18.40	+1740.0
PZT-filled silicone resin	2.87 ± 0.05	−99.8	−0.08 ± 0.003	−99.9	−0.08 ± 0.003	−99.9	0.31 ± 0.05	−99.9				
Composite lattice	74 ± 23	−93.8	−2120.10 ± 160	+1414.3	−2650.20 ± 216	+1793.0	37.00 ± 6.6	−88.6	29.00	+2800.0	69.40	+6840.0
Auxetic PZT lattice [2]			−4240.00	+2928.6	−4060.00	+2800.0	400.00	0.0	30.30	+2930.0	29.00	+2800.0

## Data Availability

Not applicable.

## Supplementary Materials

The following supporting information can be downloaded at: https://www.mdpi.com/article/10.3390/ma15227893/s1.
