# Supplementary material for: Improved Mechanical Amplification of Monolithic PZT and PZT Composite via Optimized Honeycomb Macrostructures
Source: Materials (Basel). 2022 Nov 8;15(22):7893. doi: 10.3390/ma15227893 (PMC9717726; doi:10.3390/ma15227893)
Supplement: Supplementary file 1 [file materials-15-07893-s001.zip › materials-1983881-Supplementary.pdf]

S.0. In General

The mechanical amplification of the bulk reference is 1 independent of the excitation (compression or electrical field) and the measured direction X- or Y, Equation (S1).

$$a_{x,y}^{bulk,mech} = \frac{\varepsilon_{x,y}^{reference}}{\varepsilon_{x,y}^{reference}} = 1 \quad \text{(S1)}$$

S.1 Mechanical calculations

The calculated and measured Young's modulus and, compressive strength of PZT bulk material, PZT lattices, silicone resin, PZT filled silicon resin and the composite lattices consisting of PZT building blocks and PZT filled silicone resin were mentioned in Table 2.

Table S1. Measured and calculated mechanical properties of the bulk PZT, PZT lattice, and composite lattice [2, 15, 16]. In addition, the relative percentage changes of the lattice structures in relation to the bulk material are given as %. The mechanical amplification was calculated using the average of the determined deformation. Therefore, no standard deviation is given.

**Material**	**Compressive strength**		**Young's modulus *Y***		**Mechanical amplification *according Equation (S4)***	
Unit	***MPa***	*%*	*GPA*	*%*	$\lvert a_y^{mech} \rvert$	*%*
Bulk PZT	100.74±0.65		63.00±9.0			
PZT lattice	14.90±0.81	-85.2	6.33±1.3	-89.9	12	1100.0 %
Composite lattice as PZT building blocks, PZT filled silicone resin and cavities	5.17±0.73	-94.9	33.32±3.5	-47.1	73	7200.0 %

The PZT lattices had a compressive strength 14.90 MPa. That's about 14.8 % of the PZT bulk material. This value agrees with the theoretical calculation according to Equation (S2). Here, a theoretical strength of 11.65-16.16 MPa was obtained for PZT lattice structures.

$$\frac{E^{lattice}}{E^{bulk}} = \left(\frac{\rho^{lattice}}{\rho^{bulk}}\right)^2 \rightarrow E^{lattice} = \left(\frac{\rho^{lattice}}{\rho^{bulk}}\right)^2 \cdot E^{bulk} \quad \text{(S2)}$$

The composite lattice consisting of PZT building blocks and PZT filled silicone resin reached a compressive strength of 5.17 MPa

The mechanical $\varepsilon^{reference,mech}$ was obtained from the fracture load of the composite lattice or the PZT lattice divided by the Young's modulus of the bulk PZT, Equation (S3). This means the deformation of the ceramic was calculated as if it had fractured under the same fracture load as the lattices.

$$\varepsilon_b^{reference,mech} = \frac{\sigma_b^{lattice}}{E_{PZT}} \quad \text{(S3)}$$

The strain amplification a_y^{mech} is obtained from the lattice deformation to the comparison deformation, Equation (S4) and Figure 5.

$$a_y^{mech} = \frac{\varepsilon_y^{lattice}}{\varepsilon^{reference}} = \varepsilon_y^{lattice} \cdot \frac{\sigma_b^{lattice}}{E_{PZT}} \quad \text{(S4)}$$

From the strain amplification calculations, a higher strain amplification in the Y-direction results for both composites and PZT grids compared to dense material.

S.2 Piezoelectric calculations

The piezoelectric deformation $\varepsilon_y^{lattice,piezo}$ was determined by measuring the centre unit cell geometry before and after piezoelectric excitation according to [5]. Therefore, the mechanical amplification a_y^{piezo} caused by applied electric field is defined as ratio of $\varepsilon_y^{lattice,piezo}$ to deformation of bulk material and $\rho_{relative}$, given by Equation (S5), Figure 5 and used in [2, 15, 16, 33].

$$a_{x,y}^{piezo} = \frac{\varepsilon_{x,y}^{lattice}}{\varepsilon_{x,y}^{dense}} \cdot \frac{1}{\rho_{relative}} \tag{S5}$$

ε_y^{dense} is the deformation of the material without structural influence which is represented by $\varepsilon_y^{lattice,piezo}$.

The values of the reference $d_{32}^{reference}$ as properties of the bulk material were determined according to $\varepsilon^{PZT} = d_{32}^{PZT} \cdot E_3$ ($d_{32}^{PZT}=-140$ pC N^{-1}). This results in a strain amplification in Y-direction of 18.4 for PZT lattices and of 69.4 for composite lattices.

References

2. Fey, T.; Eichhorn, F.; Han, G.; Ebert, K.; Wegener, M.; Roosen, A.; Kakimoto, K.; Greil, P. Mechanical and electrical strain response of a piezoelectric auxetic PZT lattice structure. *Smart Mater. Struct.* **2016**, *25*, 15017. https://doi.org/10.1088/0964-1726/25/1/015017.

5. Iyer, S.; Alkhader, M.; Venkatesh, T.A.; Landis, C. Electromechanical Response of Piezoelectric Honeycomb Foam Structures. *J. Am. Ceram. Soc.* **2014**, *97*, 826–834.

15. Eichhorn, F.; Keppner, F.; Köllner, D.; Fey, T. Deformation Behavior of 2D Composite Cellular Lattices of Ceramic Building Blocks and Epoxy Resin. *Adv. Eng. Mater.* **2021**, *24*, 2100536.

16. Eichhorn, F.; Schiegerl, H.; Köllner, D.; Kakimoto, K.; Fey, T. Stress and Deformation Behavior of 2D Composite Cellular Actuator Structures of Ceramic Building Blocks and Epoxy Resin. *Phys. Status Solidi B Basic Res.* **2022**, 259, 2100591. *https://*doi.org/10.1002/pssb.202100591.

33. Eichhorn, F.; Keppner, F.; Köllner, D.; Fey, T. Deformation Behavior of 2D Composite Cellular Lattices of Ceramic Building Blocks and Epoxy Resin. *Adv. Eng. Mater.* **2022**, *24*, 2270004. https://doi.org/10.1002/adem.202270004.
